# Supplementary material for: Effect of Dietary Supplements with ω-3 Fatty Acids, Ascorbic Acid, and Polyphenolic Antioxidant Flavonoid on Gene Expression, Organ Failure, and Mortality in Endotoxemia-Induced Septic Rats
Source: Antioxidants (Basel). 2023 Mar 7;12(3):659. doi: 10.3390/antiox12030659 (PMC10044831; doi:10.3390/antiox12030659)

Supplementary Information

Effect of dietary ω-3 fatty acids, ascorbic acid, and polyphenolic antioxidant flavonoid supplements on gene expression, organ failure and mortality in endotoxemic-induced septic rats.

Yolanda Prado, Cesar Echeverría, Carmen G. Feijóo, Claudia A. Riedel, Claudio Cabello-Verrugio, Juan F. Santibanez and Felipe Simon.

SUPPLEMENTARY MATERIAL AND METHODS

Protein expression determination of RMEC by flow cytometry

Flow cytometry analysis was performed to determine protein expression changes of VE-Cad, α-SMA, Col III, TNF-α, and NF-κB using corresponding monoclonal antibodies (all from R&D Systems, Inc), coupled to suitable secondary antibodies conjugated to fluorophores (all from ThermoFisher). The labeled cells were then analyzed immediately by flow cytometry (BD FACS Fortessa, BD Biosciences, San José, CA). Color compensation matrices were calculated for each staining combination within each experiment using single-stained antibody. In all analyses, doublets and clusters were eliminated. A minimum of 10,000 events were analyzed.

Statistical analyses

The relationships between variables were assessed by means of correlation analysis using Spearman's correlation coefficients and linear regression. The data were analyzed with GraphPad Prism version 9.4 (GraphPad Software, LLC). Samples used in the study were defined to identify the mean magnitude effect of ≥ 2-fold change with standard deviations of 10%. Accordingly, a sample size of 12 rats per groups, would provide 90% statistical power using a two-sided 0.05 significance level.

SUPPLEMENTARY TABLE LEGENDS

Supplementary Table S1. Correlation analyses between mRNA expression of EndMT genes and increased permeability in the mesentery, in endotoxemic condition. r^2: correlation coefficient and *p*: p-value.

Supplementary Table S2. Correlation analyses between mRNA expression of EndMT genes and increased permeability in the liver, in endotoxemic condition. r^2: correlation coefficient and *p*: p-value.

Supplementary Table S3. Correlation analyses between mRNA expression of EndMT genes and increased permeability in the kidney, in endotoxemic condition. r^2: correlation coefficient and *p*: p-value.

Supplementary Table S4. Summary of actions of dietary ω-3 fatty acids, ascorbic acid, and polyphenolic antioxidant flavonoid supplements on gene expression, organ failure and mortality in endotoxemic-induced septic rats.

SUPPLEMENTARY FIGURE LEGENDS

Supplementary Figure S1. Impact on EndMT protein expression through dietary supplementation based on ω-3 FA, ascorbic acid or polyphenolic antioxidant flavonoids in endotoxemic rats. (**A**-**D**) The mRNA expression of EndMT markers (endothelial marker VE-Cadherin (red bars) and fibrotic and ECM markers α-SMA and Coll III (green bars)), EndMT inducer TNF-α (green bars) and EndMT signaling transcription factor NF-κB (blue bars) were detected in rats subjected to endotoxemia and supplemented with ω-3 fatty acid (ω-3 FA, **A** and **B**), ascorbic acid (AsA, **C** and **D**) or polyphenolic antioxidant flavonoids (Flav, **E** and **F**) through a therapeutic (**A, C** and **E**) and a prophylactic (**B, D** and **F**) protocol. (**G**-**L**) Survival percentage was determined 7 days after endotoxemia induction in rats subjected to endotoxemia and supplemented with ω-3 fatty acid (ω-3 FA, **G** and **H**), ascorbic acid (AsA, **I** and **J**) or polyphenolic antioxidant flavonoids (Flav, **K** and **L**) through a therapeutic (**G, I** and **K**) and a prophylactic (**H, J** and **L**) protocol. Statistical differences were assessed by a one-way analysis of variance (ANOVA) (Kruskal–Wallis) followed by Dunn's *post hoc* test. *$p < 0.05$, **$p < 0.01$, ***$p < 0.001$ compared with the saline-treated condition. Determination of protein expression was normalized relative to β-tubulin expression and are expressed relative to sham-supplemented condition. Values are expressed as the mean ± SD. (N = 12).

Supplementary Table S1

Mesentery permeability in endotoxemic rats

(Evans Blue dye (ng/mg))

mRNA expression *(Log2 (fold change))*

		prophylactic ⇩ *therapeutic*	sham ⇩ sham		sham ⇩ ω-3FA		ω-3FA ⇩ ω-3FA		sham ⇩ AsA		AsA ⇩ AsA		sham ⇩ Flav		Flav ⇩ Flav	
			r^2	p	r^2	p	r^2	p	r^2	p	r^2	p	r^2	p	r^2	p
EndMT markers	Endothelial proteins	VE-Cad	**0.843**	**0.002**	**0.820**	**0.005**	**0.812**	**0.006**	**0.801**	**0.012**	**0.823**	**0.008**	**0.804**	**0.002**	**0.823**	**0.004**
		PECAM	0.623	0.034	0.763	0.010	0.723	0.009	**0.833**	**0.011**	0.775	0.009	0.635	0.012	0.735	0.009
		vWF	0.645	0.023	0.623	0.012	0.734	0.011	0.764	0.026	**0.801**	**0.007**	0.624	0.027	**0.812**	**0.008**
		Col IV	0.753	0.026	**0.827**	**0.002**	0.745	0.030	0.524	0.045	0.597	0.042	0.634	0.021	**0.824**	**0.011**
	Fibrotic and ECM proteins	α-SMA	**0.853**	**0.005**	**0.864**	**0.006**	**0.867**	**0.006**	**0.832**	**0.005**	**0.826**	**0.007**	**0.821**	**0.006**	**0.888**	**0.004**
		SM22α	0.684	0.023	0.734	0.023	0.742	0.022	0.753	0.014	0.763	0.003	0.724	0.021	0.423	0.108
		FSP-1	**0.824**	**0.003**	0.724	0.064	**0.832**	**0.023**	0.628	0.023	**0.812**	**0.012**	0.535	0.086	0.684	0.044
		Col I	0.712	0.032	0.702	0.021	**0.821**	**0.007**	0.636	0.033	**0.831**	**0.025**	0.603	0.034	0.762	0.048
		Col III	**0.821**	**0.003**	**0.808**	**0.004**	**0.814**	**0.006**	**0.834**	**0.007**	**0.845**	**0.008**	**0.863**	**0.008**	**0.835**	**0.004**
		FN	0.734	0.036	0.745	0.021	0.636	0.022	0.574	0.052	0.652	0.015	0.425	0.104	7.352	0.018
EndMT inducers		IL-1β	0.634	0.021	0.693	0.032	0.685	0.034	0.625	0.023	0.734	0.015	0.636	0.034	0.424	0.121
		IL-6	0.735	0.023	0.753	0.022	0.734	0.045	0.536	0.055	0.634	0.010	0.735	0.021	0.635	0.037
		TNF-α	**0.825**	**0.034**	**0.814**	**0.006**	**0.842**	**0.010**	**0.824**	**0.009**	**0.864**	**0.005**	**0.853**	**0.010**	**0.912**	**0.002**
		TGF-β1	0.636	0.045	0.746	0.024	0.536	0.052	0.536	0.034	0.536	0.580	0.523	0.041	0.724	0.027
		TGF-β2	0.536	0.051	0.723	0.015	0.743	0.023	0.423	0.127	0.585	0.056	0.423	0.098	**0.845**	**0.043**
EndMT signaling	Receptors	IL1R	**0.842**	**0.004**	0.734	0.011	**0.826**	**0.003**	0.734	0.021	**0.842**	**0.003**	0.653	0.037	0.724	0.023
		IL6R	0.765	0.022	0.786	0.032	0.783	0.032	0.535	0.057	0.712	0.018	0.646	0.043	0.635	0.043
		TNFR	0.698	0.023	0.647	0.042	0.742	0.004	0.634	0.010	0.685	0.021	0.647	0.034	**0.824**	**0.005**
		TβRI	0.774	0.021	0.542	0.053	0.636	0.032	0.425	0.245	0.535	0.075	0.636	0.045	0.746	0.026
		TβRII	0.686	0.043	0.577	0.046	0.684	0.031	0.486	0.174	0.596	0.067	0.643	0.041	0.735	0.021
	Transcription factors	NF-κB	**0.824**	**0.007**	**0.802**	**0.007**	**0.875**	**0.002**	**0.823**	**0.005**	**0.821**	**0.003**	**0.834**	**0.008**	**0.874**	**0.009**
		Smad4	**0.802**	**0.010**	0.746	0.009	**0.807**	**0.008**	0.654	0.047	0.533	0.026	0.745	0.012	0.635	0.026
		Slug	**0.806**	**0.005**	0.775	0.021	0.735	0.008	0.685	0.026	0.643	0.021	0.723	0.008	**0.801**	**0.005**
		Twist	0.753	0.008	0.720	0.014	0.723	0.005	0.698	0.021	0.585	0.044	0.742	0.011	0.702	0.008
		Snail	0.798	0.004	0.635	0.047	0.646	0.035	0.497	0.102	0.573	0.048	0.634	0.019	0.612	0.034
		Zeb-1	**0.832**	**0.007**	0.684	0.044	0.635	0.034	0.553	0.086	0.578	0.047	0.647	0.021	0.621	0.041
	ROS enzymes	NOX-1	0.435	0.224	0.325	0.284	0.224	0.533	0.475	0.285	0.474	0.446	0.412	0.523	0.353	0.224
		NOX-2	0.723	0.034	0.712	0.011	0.646	0.023	0.634	0.036	0.774	0.032	0.653	0.023	0.474	0.103
		NOX-4	0.423	0.335	0.446	0.274	0.436	0.324	0.424	0.425	0.335	0.536	0.523	0.324	0.536	0.475

Supplementary Table S2

Liver permeability in endotoxemic rats

(Evans Blue dye (ng/mg))

mRNA expression *(Log2 (fold change))*

		prophylactic ⇩ therapeutic	sham ⇩ sham		sham ⇩ ω-3FA		ω-3FA ⇩ ω-3FA		sham ⇩ AsA		AsA ⇩ AsA		sham ⇩ Flav		Flav ⇩ Flav	
			r^2	p	r^2	p	r^2	p	r^2	p	r^2	p	r^2	p	r^2	p
EndMT markers	Endothelial proteins	VE-Cad	**0.853**	**0.007**	**0.804**	**0.004**	**0.864**	**0.003**	**0.853**	**0.004**	**0.864**	**0.004**	**0.843**	**0.003**	**0.812**	**0.008**
EndMT markers	Endothelial proteins	PECAM	0.745	0.017	0.722	0.041	**0.846**	**0.004**	**0.821**	**0.002**	**0.853**	**0.004**	0.664	0.014	**0.801**	**0.008**
EndMT markers	Endothelial proteins	vWF	0.698	0.032	0.698	0.049	0.744	0.022	**0.802**	**0.007**	0.746	0.004	0.675	0.018	0.753	0.012
EndMT markers	Endothelial proteins	Col IV	0.724	0.021	0.753	0.036	0.646	0.048	0.643	0.046	0.636	0.041	0.646	0.031	0.732	0.023
EndMT markers	Fibrotic and ECM proteins	α-SMA	**0.842**	**0.004**	**0.864**	**0.007**	**0.864**	**0.006**	**0.853**	**0.005**	**0.825**	**0.005**	**0.806**	**0.002**	**0.824**	**0.005**
EndMT markers	Fibrotic and ECM proteins	SM22α	**0.842**	**0.006**	0.721	0.032	**0.857**	**0.003**	**0.845**	**0.005**	0.637	0.024	0.773	0.043	0.735	0.108
EndMT markers	Fibrotic and ECM proteins	FSP-1	**0.817**	**0.005**	0.774	0.048	**0.835**	**0.006**	0.646	0.510	0.753	0.023	0.685	0.045	0.634	0.034
EndMT markers	Fibrotic and ECM proteins	Col I	0.634	0.045	**0.808**	**0.004**	**0.854**	**0.003**	0.735	0.511	**0.864**	**0.034**	0.745	0.019	0.628	0.046
EndMT markers	Fibrotic and ECM proteins	Col III	**0.864**	**0.008**	**0.853**	**0.005**	**0.847**	**0.007**	**0.863**	**0.006**	**0.852**	**0.009**	**0.808**	**0.004**	**0.823**	**0.002**
EndMT markers	Fibrotic and ECM proteins	FN	0.674	0.043	0.747	0.17	**0.864**	**0.003**	0.657	0.271	0.736	0.028	0.545	0.164	**0.865**	**0.003**
EndMT inducers		IL-1β	0.654	0.031	0.743	0.025	**0.831**	**0.025**	0.746	0.31	0.732	0.043	0.745	0.017	0.545	0.041
EndMT inducers		IL-6	0.636	0.041	0.635	0.032	0.675	0.034	0.675	0.042	0.753	0.021	0.712	0.014	0.546	0.045
EndMT inducers		TNF-α	**0.845**	**0.006**	**0.821**	**0.004**	**0.802**	**0.007**	**0.827**	**0.004**	**0.857**	**0.003**	**0.835**	**0.003**	**0.845**	**0.003**
EndMT inducers		TGF-β1	0.674	0.036	0.690	0.032	0.547	0.185	0.623	0.042	0.636	0.032	0.635	0.047	0.646	0.076
EndMT inducers		TGF-β2	0.612	0.042	0.775	0.009	0.528	0.123	0.546	0.164	0.585	0.041	0.546	0.232	0.536	0.051
EndMT signaling	Receptors	IL1R	**0.832**	**0.006**	0.743	0.023	0.747	0.024	0.652	0.164	**0.825**	**0.023**	0.753	0.024	0.735	0.034
EndMT signaling	Receptors	IL6R	0.698	0.016	0.734	0.018	**0.863**	**0.008**	0.754	0.076	0.753	0.015	0.710	0.019	0.743	0.043
EndMT signaling	Receptors	TNFR	0.735	0.025	0.763	0.037	**0.844**	**0.002**	0.523	0.274	0.735	0.024	0.764	0.024	0.724	0.024
EndMT signaling	Receptors	TβRI	0.586	0.052	0.634	0.034	0.735	0.085	0.525	0.253	0.628	0.023	0.724	0.023	**0.846**	**0.003**
EndMT signaling	Receptors	TβRII	0.596	0.580	0.624	0.032	0.636	0.143	0.567	0.324	0.535	0.045	0.647	0.042	**0.854**	**0.004**
EndMT signaling	Transcription factors	NF-κB	**0.842**	**0.004**	**0.832**	**0.005**	**0.864**	**0.001**	**0.853**	**0.003**	**0.815**	**0.004**	**0.834**	**0.003**	**0.843**	**0.005**
EndMT signaling	Transcription factors	Smad4	**0.864**	**0.003**	0.724	0.013	**0.807**	**0.003**	0.763	0.042	**0.811**	**0.023**	0.723	0.032	0.685	0.034
EndMT signaling	Transcription factors	Slug	0.753	0.078	0.746	0.035	**0.802**	**0.005**	**0.835**	**0.002**	0.843	0.004	0.754	0.042	**0.832**	**0.006**
EndMT signaling	Transcription factors	Twist	**0.824**	**0.006**	**0.823**	**0.003**	0.642	0.632	0.735	0.033	0.734	0.032	0.742	0.034	0.708	0.003
EndMT signaling	Transcription factors	Snail	**0.835**	**0.008**	0.735	0.414	0.753	0.032	0.585	0.153	0.698	0.038	0.635	0.051	0.536	0.046
EndMT signaling	Transcription factors	Zeb-1	**0.835**	**0.009**	0.692	0.053	0.732	0.043	0.532	0.065	0.654	0.042	0.546	0.075	0.586	0.042
EndMT signaling	ROS enzymes	NOX-1	0.425	0.367	0.374	0.375	0.353	0.364	0.374	0.296	0.536	0.275	0.535	0.343	0.435	0.453
EndMT signaling	ROS enzymes	NOX-2	0.794	0.021	0.734	0.021	0.725	0.012	0.764	0.027	**0.853**	**0.007**	0.744	0.034	0.743	0.027
EndMT signaling	ROS enzymes	NOX-4	0.512	0.275	0.375	0.353	0.374	0.243	0.374	0.346	0.475	0.335	0.524	0.243	0.635	0.335

Supplementary Table S3

kidney permeability in endotoxemic rats

(Evans Blue dye (ng/mg))

mRNA expression (*Log2 (fold change)*)		*prophylactic* ⇩ *therapeutic*	sham ⇩ sham		sham ⇩ ω-3FA		ω-3FA ⇩ ω-3FA		sham ⇩ AsA		AsA ⇩ AsA		sham ⇩ Flav		Flav ⇩ Flav	
			r^2	p	r^2	p	r^2	p	r^2	p	r^2	p	r^2	p	r^2	p
EndMT markers	Endothelial proteins	VE-Cad	**0.813**	**0.009**	**0.853**	**0.007**	**0.842**	**0.002**	**0.813**	**0.006**	**0.823**	**0.003**	**0.832**	**0.004**	**0.823**	**0.006**
EndMT markers	Endothelial proteins	PECAM	0.735	0.14	0.754	0.025	**0.814**	**0.003**	**0.804**	**0.008**	**0.831**	**0.005**	0.745	0.011	0.703	0.038
EndMT markers	Endothelial proteins	vWF	0.743	0.16	0.536	0.043	**0.834**	**0.031**	0.735	0.023	0.723	0.032	0.634	0.031	**0.843**	**0.006**
EndMT markers	Endothelial proteins	Col IV	0.757	0.15	0.647	0.023	0.753	0.023	0.712	0.056	0.721	0.024	0.628	0.023	0.754	0.034
EndMT markers	Fibrotic and ECM proteins	α-SMA	**0.843**	**0.005**	**0.821**	**0.003**	**0.853**	**0.006**	**0.813**	**0.003**	**0.821**	**0.003**	**0.812**	**0.004**	**0.864**	**0.001**
EndMT markers	Fibrotic and ECM proteins	SM22α	**0.814**	**0.007**	**0.842**	**0.007**	**0.876**	**0.003**	**0.821**	**0.002**	0.734	0.034	0.743	0.043	0.742	0.047
EndMT markers	Fibrotic and ECM proteins	FSP-1	**0.822**	**0.003**	0.742	0.017	**0.824**	**0.006**	0.623	0.232	**0.832**	**0.002**	0.712	0.047	0.697	0.041
EndMT markers	Fibrotic and ECM proteins	Col I	0.753	0.034	0.764	0.023	0.753	0.006	0.653	0.423	0.702	0.045	0.624	0.041	**0.843**	**0.006**
EndMT markers	Fibrotic and ECM proteins	Col III	**0.842**	**0.003**	**0.824**	**0.002**	**0.846**	**0.007**	**0.823**	**0.005**	**0.812**	**0.007**	**0.834**	**0.002**	**0.803**	**0.003**
EndMT markers	Fibrotic and ECM proteins	FN	0.721	0.034	**0.815**	**0.004**	**0.823**	**0.003**	0.694	0.150	0.701	0.032	0.635	0.059	0.745	0.043
EndMT inducers		IL-1β	0.665	0.031	0.713	0.012	**0.853**	**0.003**	0.702	0.037	0.712	0.032	**0.832**	**0.002**	0.624	0.021
EndMT inducers		IL-6	0.745	0.024	0.743	0.010	0.742	0.031	0.643	0.039	0.701	0.023	**0.875**	**0.001**	0.657	0.042
EndMT inducers		TNF-α	**0.864**	**0.008**	**0.813**	**0.006**	**0.843**	**0.004**	**0.823**	**0.002**	**0.822**	**0.004**	**0.823**	**0.004**	**0.812**	**0.006**
EndMT inducers		TGF-β1	0.756	0.024	0.535	0.143	0.634	0.243	0.732	0.021	0.612	0.047	0.745	0.032	0.536	0.054
EndMT inducers		TGF-β2	0.626	0.45	0.723	0.087	0.646	0.353	0.624	0.244	0.633	0.038	0.632	0.045	0.684	0.067
EndMT signaling	Receptors	IL1R	**0.846**	**0.005**	0.735	0.032	0.742	0.018	0.732	0.032	0.742	0.032	0.723	0.034	0.793	0.041
EndMT signaling	Receptors	IL6R	0.753	0.021	0.712	0.039	0.635	0.021	**0.842**	**0.003**	0.767	0.022	0.624	0.045	**0.821**	**0.006**
EndMT signaling	Receptors	TNFR	0.646	0.032	0.756	0.041	0.754	0.026	0.643	0.046	**0.834**	**0.005**	0.734	0.037	**0.902**	**0.002**
EndMT signaling	Receptors	TβRI	0.635	0.028	0.748	0.043	0.642	0.038	0.597	0.142	0.745	0.034	0.634	0.047	**0.843**	**0.004**
EndMT signaling	Receptors	TβRII	0.685	0.034	0.623	0.053	0.746	0.033	0.643	0.243	0.646	0.040	0.697	0.049	0.723	0.023
EndMT signaling	Transcription factors	NF-κB	**0.853**	**0.003**	**0.825**	**0.007**	**0.824**	**0.003**	**0.832**	**0.002**	**0.804**	**0.005**	**0.823**	**0.005**	**0.854**	**0.007**
EndMT signaling	Transcription factors	Smad4	**0.854**	**0.002**	0.765	0.010	0.745	0.034	0.734	0.023	0.744	0.032	0.723	0.043	0.732	0.041
EndMT signaling	Transcription factors	Slug	**0.824**	**0.003**	0.643	0.042	**0.844**	**0.004**	0.753	0.021	0.795	0.012	0.721	0.038	**0.832**	**0.005**
EndMT signaling	Transcription factors	Twist	**0.864**	**0.002**	0.647	0.044	**0.864**	**0.002**	0.744	0.026	**0.832**	**0.004**	0.657	0.043	0.634	0.041
EndMT signaling	Transcription factors	Snail	0.753	0.015	0.753	0.021	0.647	0.040	0.634	0.046	0.753	0.022	0.686	0.048	0.684	0.042
EndMT signaling	Transcription factors	Zeb-1	**0.832**	**0.006**	0.785	0.015	0.624	0.048	0.647	0.048	0.634	0.054	0.676	0.049	0.623	0.047
EndMT signaling	ROS enzymes	NOX-1	0.524	0.285	0.435	0.453	0.536	0.364	0.435	0.321	0.435	0.285	0.634	0.104	0.474	0.224
EndMT signaling	ROS enzymes	NOX-2	**0.853**	**0.007**	0.745	0.032	**0.834**	**0.002**	0.735	0.023	**0.845**	**0.006**	0.721	0.038	**0.834**	**0.004**
EndMT signaling	ROS enzymes	NOX-4	0.684	0.087	0.536	0.078	0.446	0.007	0.495	0.335	0.423	0.242	0.635	0.098	0.423	0.243

Supplementary Table S4

Supplementary Table S4. Summary of actions of dietary ω-3 fatty acids, ascorbic acid, and polyphenolic antioxidant flavonoid supplements on gene expression, organ failure and mortality in endotoxemic-induced septic rats**.**

		Supplementation:	**Sham**	**ω-3 Fatty acid**		**Ascorbid Acid**		**Flavonoid**	
		Protocol:		Therapeutic	Prophylactic	Therapeutic	Prophylactic	Therapeutic	Prophylactic
**Gene expression**	**EndMT markers**	VE-Cad	**	*	NS	**	*	*	NS
		PECAM	**	NS	NS	**	*	*	NS
		vWF	**	NS	NS	**	*	*	NS
		Col IV	***	*	*	**	*	*	NS
		α-SMA	***	*	NS	***	*	**	NS
		SM22α	***	*	*	***	*	*	*
		FSP-1	***	NS	NS	*	*	*	NS
		Col I	***	NS	NS	*	*	**	NS
		Col III	***	NS	NS	***	**	***	NS
		FN	***	NS	NS	***	**	***	NS
	**EndMT inducers**	IL-1β	***	NS	NS	***	**	***	NS
		IL-6	**	NS	NS	*	*	*	NS
		TNF-α	***	*	*	**	*	*	*
		TGF-β1	***	NS	NS	**	*	**	NS
		TGF-β2	**	NS	NS	*	*	*	NS
	**EndMT signaling**	IL1R	***	*	NS	**	***	***	NS
		IL6R	***	*	NS	**	***	***	NS
		TNFR	***	**	*	***	***	***	*
		TβRI	*	*	NS	NS	NS	NS	NS
		TβRII	NS	NS	NS	NS	NS	NS	NS
		NF-κB	***	**	NS	***	***	**	NS
		Smad4	***	**	NS	***	***	**	NS
		Slug	***	**	NS	***	***	**	NS
		Twist	***	*	NS	**	***	**	NS
		Snail	***	*	NS	***	***	**	NS
		Zeb-1	***	**	NS	**	*	**	NS
		NOX-1	NS	NS	NS	NS	NS	NS	NS
		NOX-2	***	**	*	**	*	*	*
		NOX-4	NS	NS	NS	NS	NS	NS	NS
**Vascular permeability**		Mesentery	***	**	*	***	**	**	**
		Liver	***	**	*	***	**	***	**
		Kidney	***	**	NS	***	***	***	**
**Non-survivor Vascular permeability**		Mesentery	*	*	*	NS	NS	NS	*
		Liver	*	*	*	NS	NS	NS	*
		Kidney	*	*	*	NS	NS	NS	*
**Systolic pressure**			***	***	*	***	***	***	**
**Heart rate**			***	**	NS	***	***	***	**
**MODS**		ALT	***	**	NS	***	***	***	**
		AST	***	**	*	***	***	***	**
		TBIL	***	**	*	***	***	***	***
		GGT	***	**	NS	***	***	**	*
		Lac	***	**	*	***	***	***	***
		CRE	***	**	*	**	**	**	*
		BUN	***	**	*	**	**	**	**
		BUN/CRE	***	***	NS	***	***	***	***
		eGFR	**	**	NS	**	**	**	**
		Gly	**	*	NS	**	**	**	**
**Mortality at 7 days**			75 %	50 %	17 %	75 %	67 %	67 %	50 %
**Risk of death**			‡‡‡	NS	NS	‡‡‡	‡‡	‡‡	NS

*: p-value < 0.05; **: p-value < 0.01; ***: p-value < 0.001; ‡: p-value < 0.05; ‡‡: p-value < 0.01; NS: non-significant change. All compared with saline-treated rats.

Supplementary Figure S1

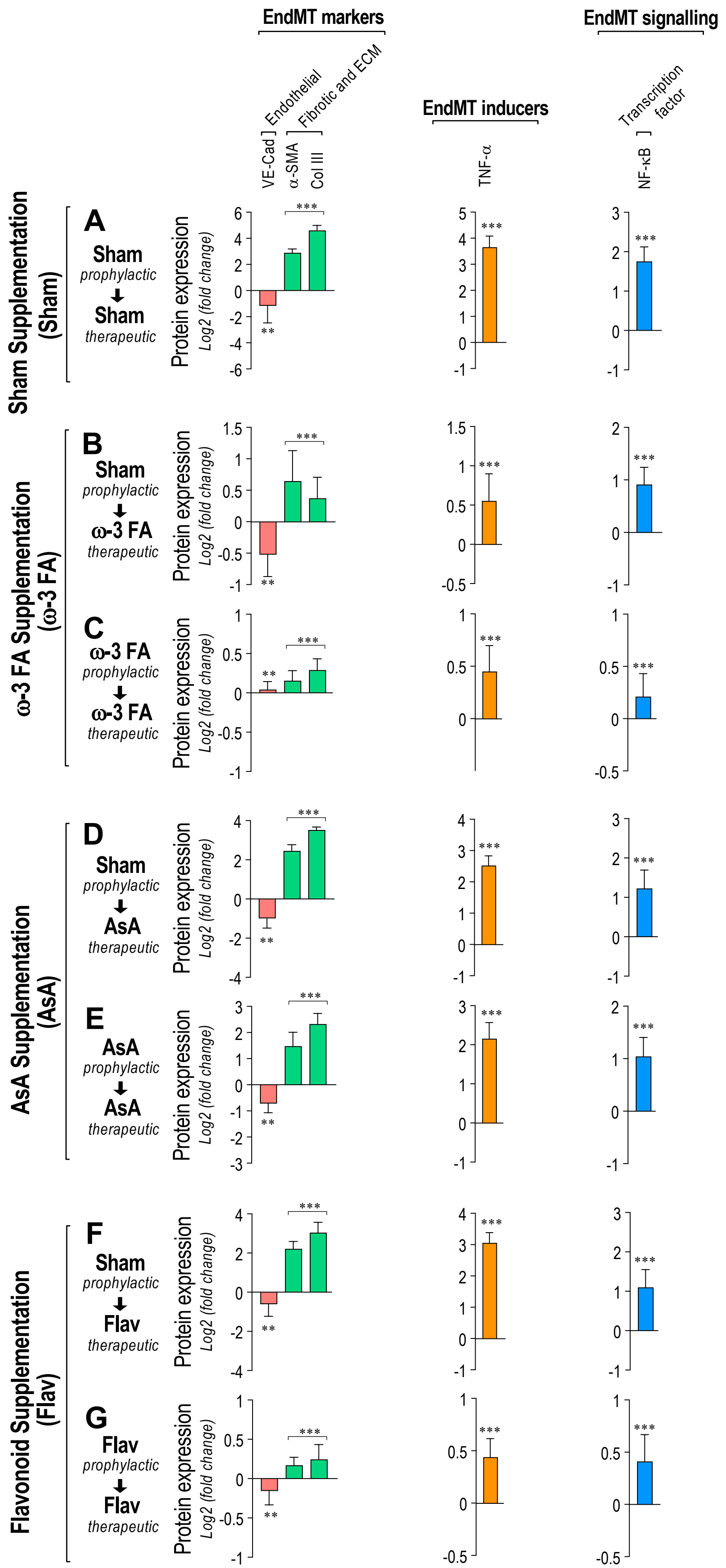

Supplement: Supplementary file 1 [file antioxidants-12-00659-s001.zip › antioxidants-2239645-supplementary.pdf]
